# Ending one conversation, starting another

**DOI:** 10.1172/JCI193004

**Published:** 2025-04-01

**Authors:** Ushma S. Neill

With this entry, the Conversations with Giants in Medicine series comes to a close. The *JCI* is heading in a new direction with its video content, and I too will be heading in a different career direction that makes it hard to continue the series with the same care. As is often noted, “All good things must come to an end.” To watch a supercut of the last question I asked each subject, “What other vocation could have kept you as motivated?” see the *JCI* website at https://www.jci.org/videos/cgms.

Since the Conversations series was first pitched as an idea by Howard Rockman when he took over as *JCI* Editor in Chief in 2012, it has spanned 74 interviews with 80 people. The interviewees included 15 Nobel Prize laureates, 27 Lasker Prize winners, 65 members of the National Academy of Sciences, and 70 members of the National Academy of Medicine. Eighteen of the interviewees held two X chromosomes, and the remaining 62 were men. Gratifyingly, we got to hear the stories of nine legendary scientists before they passed away (Paul Greengard, Paul Marks, Bill Paul, Don Seldin, Lloyd Holly Smith, Oliver Smithies, Tom Starzl, Jean Wilson, and Tachi Yamada).

The pandemic resulted in a shift of 14 of the interviews to a virtual format, with the other 60 in person. Five of the interviews were conducted in pairs or trios, which led to a faster pace of questioning and often induced more laughter; those who work in close partnership often know how to bring out hilarious stories from one another. I recall Howard Rockman noting that watching Bob Lefkowitz interview Joe Goldstein and Mike Brown was, “akin to watching Babe Ruth interview Joe DiMaggio and Lou Gehrig.” He, as an avowed hockey fan, however, does not recall making this reference! In another instance, I got to watch as Sir Marc Feldmann interviewed Jacques Miller and Max Cooper. I felt like I got to watch a master class in immunology.

When a colleague downloaded data on viewership for me back in January 2025, we noted that the collective videos have clocked over 256,000 views, with a total watch time of 37,411 hours (over 4.25 years!). It is beyond gratifying to know that more of you than just my parents watched these videos. The July 2014 interview with Tony Fauci was watched over 54,000 times and even got me quoted in a profile on Dr. Fauci in the *New Yorker*. I think more people sent me that link than any other coverage I’ve gotten.

The series launched exactly 13 years ago, when colleagues convinced Harold Varmus to be our first subject. I tried so hard to be prepared and had read tens of interviews and his myriad opinion pieces, and yet still when I asked him one question midway through the interview, his reply was, “Why don’t I answer the question I think you should have asked me?” That bit was carefully edited out.

I perhaps got better at being even MORE prepared and better able to steer the conversations. Don Ganem came close to calling me a cyber stalker for asking him about getting an F in eighth grade algebra for holding hands with the daughter of his school’s headmaster. But truly, part of the fun was uncovering the layers of people’s stories, hearing about their early influences, through their training and establishment of their labs, and sometimes their political leanings. The one throughline in all the interviews was the intense passion for science and the thrill of discovery.

I also always asked each subject the same last question, “What other vocation do you think could have kept you as motivated, if you could not have been a scientist or physician?” So many pushed back and wondered how there could ever be another career (see Kathy High’s reply to the question at the 37-minute mark in her interview), but in our midst are those who were almost teachers, lawyers, stand-up comedians, historians, or politicians. As I’ve now had a career spanning science, editorial, and research and educational administration, I think my alternate career is to be a pastry chef; there is something so scientific and specific and yet creative about patisserie.

Thank you Howard Rockman for the original idea for this series, and much gratitude to the producer and host of 24 of the first videos, Francesco Rulli. He also introduced me to my close colleagues Semyon Maltsev and Alexey Levchenko (Figure 1), who shot and edited 39 of the videos. They laughed and learned with me as each new subject came into the room and made me look better in the final videos. I end this 22-year run at the *JCI*, first as Executive Editor and for the last 13 years as Editor-at-Large with a full heart and such gratitude for being able to have the chance to hear (and tell) the stories of our Giants in Medicine.

## Figures and Tables

**Figure 1:**
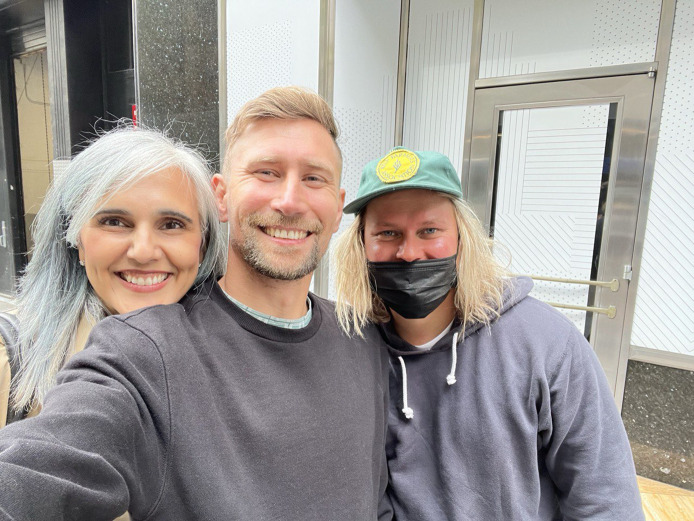
Ushma Neill, Semyon Maltsev, and Alexey Levchenko in NYC, September 2023.

## Version 1 04/01/2025

Electronic publication

